# A Cross-Sectional Study to Assess HPV Knowledge and HPV Vaccine Acceptability in Mali

**DOI:** 10.1371/journal.pone.0056402

**Published:** 2013-02-19

**Authors:** Danielle N. Poole, J. Kathleen Tracy, Lauren Levitz, Mali Rochas, Kotou Sangare, Shahla Yekta, Karamoko Tounkara, Ben Aboubacar, Ousmane Koita, Mark Lurie, Anne S. De Groot

**Affiliations:** 1 Public Health Program, Brown University, Providence, Rhode Island, United States of America; 2 Departments of Epidemiology and Public Health, University of Maryland School of Medicine, Baltimore, Maryland, United States of America; 3 GAIA Vaccine Foundation, Providence, Rhode Island, United States of America and Bamako, Mali; 4 University of Bamako, Bamako, Mali; 5 University of Rhode Island, Providence, Rhode Island, United States of America; University of Ottawa, Canada

## Abstract

Despite a high prevalence of oncogenic human papilloma virus (HPV) infection and cervical cancer mortality, HPV vaccination is not currently available in Mali. Knowledge of HPV and cervical cancer in Mali, and thereby vaccine readiness, may be limited. Research staff visited homes in a radial pattern from a central location to recruit adolescent females and males aged 12–17 years and men and women aged ≥18 years (N = 51) in a peri-urban village of Bamako, Mali. Participants took part in structured interviews assessing knowledge, attitudes, and practices related to HPV, cervical cancer, and HPV vaccination. We found low levels of HPV and cervical cancer knowledge. While only 2.0% of respondents knew that HPV is a sexually transmitted infection (STI), 100% said they would be willing to receive HPV vaccination and would like the HPV vaccine to be available in Mali. Moreover, 74.5% said they would vaccinate their child(ren) against HPV. Men were found to have significantly greater autonomy in the decision to vaccinate themselves than women and adolescents (p = 0.005), a potential barrier to be addressed by immunization campaigns. HPV vaccination would be highly acceptable if the vaccine became widely available in Bamako, Mali. This study demonstrates the need for a significant investment in health education if truly informed consent is to be obtained for HPV vaccination. Potential HPV vaccination campaigns should provide more information about HPV and the vaccine. Barriers to vaccination, including the significantly lower ability of the majority of the target population to autonomously decide to get vaccinated, must also be addressed in future HPV vaccine campaigns.

## Introduction

Human papillomavirus (HPV) infection is a common and necessary cause of cervical cancer [1]. Each year, 60 to 70% of all cervical cancer cases worldwide are the result of high risk HPV types 16 and 18, the oncogenic genotypes against which the Cervarix® and Gardasil® vaccines protect [2]–[3]. Both vaccines have been shown to be efficacious in preventing infection with HPV types 16 and 18 among adolescent females and women [4]–[6], however Gardasil® is the only available vaccine efficacious against HPV types 16 and 18 infection in males [7].

Eighty-percent of cervical cancer cases diagnosed each year occur in developing countries [8], in part due to the lack of availability of early prevention cytology-screening programs. Educational and attitudinal barriers have also been identified as major reasons for low screening prevalence in developing countries [9]–[11]. In sub-Saharan Africa, cervical cancer rates are among the highest in the world, with an age-standardized incidence rate (ASR) of 31.0 per 100,000 women [12]. The HIV epidemic is an important contributor to the cervical cancer burden in sub-Saharan Africa [13]. HIV-seropositive women are six times more likely to develop cervical cancer compared to HIV-seronegative women due to HIV-related immune suppression [14].

Other risk factors for HPV infection, and thereby the potential for subsequent disease, include tobacco smoking, high parity, and long-term hormonal contraceptive use [15]. Early age of first sexual intercourse has long been associated with an increased risk of cervical cancer, possibly due to the biological predisposition of the immature cervix during adolescence with increased susceptibility to persistent HPV infections and therefore a greater risk of cervical cancer [16]. Age at first sexual intercourse, age at first pregnancy, and age at first marriage are highly interrelated and have similar cervical cancer risk estimates [17]. Moreover, the influence of male partners, both directly and indirectly, on women’s health has been demonstrated in the risk areas of contraception, pregnancy, and childbirth [18].

Mali is a landlocked West African country with an estimated 13 million persons, 90% of whom are Muslim [19]. In Mali, high risk HPV oncogenic types 16 and 18 have a prevalence of 12% among previously unscreened women of the general population aged 15–65 years [20]–[21], with predominant subtypes HPV-16 associated with 50% and HPV-18 associated with 12.7% of cervical cancer [22]. Among Malian women, the ASR of cervical cancer is 37.7 cases per 100,000, the second highest rate of cervical cancer in the sixteen West African countries [23]. Cervical cancer is not only the most common cancer among women in Mali; it is also the leading cause of all cancer-related mortalities among Malians [24]. However, only 4.8% of women (aged 18–69 years) have ever been screened for cervical cancer in Mali and thus cervical cancer rates may be higher than reported [23]. Due to a lack of cytology screening and early treatment programs, the mortality rate among Malian women diagnosed with cervical cancer is 80%, or 1,076 deaths per year [23].

HPV vaccination offers protection against the development of cervical cancer and associated morbidities and mortalities, and is especially valuable in resource-limited settings where secondary prevention methods such as cytology screening are not widely available and may not be a cost-effective [25]. Recent work focusing on HPV vaccine acceptability in resource-limited settings including Kenya, Tanzania, Uganda, Latin America, the Caribbean, Vietnam, Malaysia, China, and India have generally found high acceptability of HPV vaccines [26]–[30]. Several studies have shown that knowledge of HPV, cervical cancer, and risk factors for cervical cancer are low among the general public, in both high and low income settings [31]–[33]. At the time of this publication, only one other study has quantitatively examined HPV vaccine acceptability in West Africa, finding similar high levels of HPV vaccine acceptability despite low levels of knowledge in Ghana [34]. In the absence of accessible cytology screening, implementation of an HPV immunization program should be a public health priority in Mali to reduce the high burden of cervical cancer-associated morbidity and mortality. Moreover, Mali is eligible to receive Global Alliance for Vaccines and Immunization (GAVI) support for a two-year demonstration program in preparation for national introduction of HPV vaccine. This support includes the ability to purchase vaccines at a discounted price and grants to build capacity for scaling up national immunization programs. A mathematical modeling study assessing the impact of a single vaccine campaign in Mali showed decreases in the prevalence of HPV infection proportionate to achieved vaccination coverage [20].

Sexually active adolescents and adults must voluntarily agree to receive the vaccine, a decision affected by individual knowledge, beliefs about susceptibility, perception of vaccine effectiveness, family and physician perspectives, sexual and cultural practices, and cost for vaccination [35]. This cross-sectional study aimed to determine HPV knowledge, acceptability, and other factors associated with the feasibility of HPV vaccine implementation in Mali. To our knowledge, this is the first West African study to include males and females aged 12 years and older in such an assessment. We assessed adult and adolescent male and female awareness and acceptance of HPV infection and vaccination for cervical cancer prevention, as well as factors associated with willingness to be administered the vaccine among individuals in Mèkin-Sikoro, a peri-urban village in Bamako, Mali.

## Methods

### Ethics Statement

This research received ethical approval from the Committee of Ethics of the Faculty of Medicine, Pharmacy, and Odonto-Stomatology at the University of Bamako, Mali and the Ethical and Independent Review Services West Coast Board, United States.

### Study Procedures

Face-to-face structured interviews were conducted in 2011 with 51 participants. All data collection activities took place prior to HPV vaccine licensing in Mali. A two-stage sampling method was utilized. Two geographical areas were selected from the peri-urban village of Mèkin-Sikoro using a convenience sampling technique according to their proximity to the central local health clinic. In each of the selected sectors, interviewers approached households starting with the nearest home to the central health clinic.

Each interviewer was sex- and age-paired with a specific sample subset; adolescent females, adolescent males, adult females, or adult males. The interviewers approached households separately such that one interviewer approached the closest household, a second interviewer approached the next closest household, a third interviewer approached the third closest household, and a fourth interviewer approached the fourth nearest household.

Households were selected to participate if an eligible participant was present at the time of visit. Adolescents were eligible if aged 12–18 years and living in the household, and a guardian was available to provide consent. Adults were eligible if aged >18 years and living in the household. If a household had more than one eligible inhabitant who could be sex- and age-matched with the household interviewer, the first eligible individual to interact with the interviewer was selected to undergo the informed consent process after an explanation of the nature of the study. The consent form was read aloud upon request and to all illiterate individuals in Bambara, the main language in Mali [36]. Illiterate individuals who chose to take part in the study completed the informed consent process by signing with a fingerprint and a literate witness verified the process with a signature.

A standardized questionnaire assessing knowledge, vaccine acceptability and willingness to participate in an HPV vaccination program, and demographic characteristics related to HPV and cervical cancer was administered. Open response questions included reasons why or why not most participants would vaccinate themselves or their children, the preferred method for contact about vaccination appointment, and the cost participants would be willing to pay to receive HPV vaccination. The structured interviews were conducted in Bambara.

Data was de-identified and analyzed using STATA 10.0 [37]. The significance level was set at p<0.05. Data analysis consisted of descriptive statistics including frequencies and mean scores for the demographic variables. Data are presented as mean (SD) for continuous variables. Fischer’s exact tests were used to examine differences found across participant subset responses.

## Results

### 1. Characteristics of the Sample

A summary of the sample characteristics is provided in Table 1. Of the total 52 eligible participants approached, 98.1% consented to participation (n = 51). The mean age of participants was 26.1 years (SD±14.58), with 49.0% of the participants adolescents aged 12–18 years. The majority of participants had received some formal schooling (68.6%) and 69.2% of adults worked outside of the home (58.3% females, 84.6% males). Of the 43.1% of participants who were married, 36.4% were in polygamous marriages with a mean of 2.25 wives (SD±0.46). Among all participants, the mean age of first marriage was 18.67 years (SD±4.03). The mean age of sexual debut was 17.0 years (SD±1.74) for females and males, and the mean number of total sexual partners was reported to be 2.06 (SD±1.87, females 1.40, SD±0.74; males 2.65, SD±2.34). The majority of participants reported being circumcised (80.8% males) or excised (Type 2 female genital cutting, 88.0% of females).

**Table 1:** Characteristics of study participants.

Characteristic	Total		Female		Male	
	Mean (N)	SD	Mean (N)	SD	Mean (N)	SD
Age: mean (SD)	26.1 (51)	14.6	24.4 (25)	10.9	27.7 (26)	17.5
Adolescents	14.1 (20)	1.6	13.8 (8)	1.5	14.3 (12)	1.8
Adults aged 18–26 years	20.2 (11)	2.6	20.4 (8)	3.0	19.7 (3)	1.5
Adults >26 years	41.4 (20)	11.6	37.4 (9)	4.8	44.6 (11)	14.5
Any school-based education	68.6%		64.0%		73.2%	
Adult working outside the home (%)	69.2% (31)		58.3% (17)		84.6% (14)	
Marital status (% married)	43.1% (51)		52.0% (25)		34.6% (26)	
Adolescents	0.0% (20)		0.0% (8)		0.0% (12)	
Adults	71.0% (31)		76.5% (17)		64.3% (14)	
Polygamous marriage (%)^1^	36.4% (22)		30.8% (13)		44.4% (9)	
Number of wives^2^	2.3	0.5	2.3	0.5	2.3	0.5
Age of first marriage	18.7	4.0	16.9	2.0	23.2	4.7
Age of sexual debut^3^	16.5 (22)	2.3	16.8 (15)	1.7	16.0 (7)	3.3
No sexual relations^4^	29.4% (15)		28.0% (7)		34.6% (8)	
Refused to answer	27.5% (14)		1.2% (3)		42.3% (11)	
>15 years (%)	40.9%		40.0%		42.9%	
≥15 and <18 years (%)	36.4%		40.0%		28.6%	
≥18 years (%)	22.7%		20.0%		28.6%	
Number of sexual partners^5^	2.1 (22)	1.9	1.4 (15)	0.7	2.7 (7)	2.3
Number of sexual partners before marriage^6^	1.0	(2.3)	0.4	0.7	1.9	3.2
Circumcised/excised^7^	84.3%		88.0%		80.8%	
Refused to answer	7.8%		0.0%		15.4%	
Sex in exchange for money or gifts	0.0%		41.7%		0.0%	

*Note. SD = standard deviation*.

1 Among those who were married (Number who were married).

2 Among those reporting being in a polygamous marriage.

3 Among those reporting having ever had sex (Number reporting ever having sex).

4 Number reporting no sexual relations.

5 Among those reporting having ever had sex (Number reporting ever having sex).

6 Of those reporting marriage.

7 Excision refers to Type II female genital cutting, or the removal of the clitoris and inner labia.

### 2. Knowledge of STIs, HPV and Cervical Cancer

Of the participants, 62.0% (68.0% females, 56.0% males) reported they knew what an STI was, and 54.9% (64.0% females, 46.2% males) could correctly identify a method of protection against STIs (Table 2). Just over half (54.9%) of participants knew of places where STI screening and treatment were offered, and 23.5% of participants reported ever having an STI (40.0% females, 7.7% males). Only one participant knew HPV was an STI. Following a brief definition of HPV, 24.0% of participants thought HPV only infects females only, 30.0% thought HPV only infects males, 56.0% thought HPV infects both males and females, and 14.0% did not know who was susceptible to HPV infection. Only 9.8% of female participants had heard of cervical cancer.

**Table 2:** Understanding of STIs [Sexually Transmitted Infections], HPV, and cervical cancer.

	Total % (N)	Female % (N)	Male % (N)
**STIs**			
Knows what a sexually transmitted infection is	60.8% (51)	68.0% (25)	53.8% (26)
Adolescents	40.0% (20)	37.5% (8)	41.7% (12)
Adults	45.1% (51)	82.4% (17)	64.3% (14)
Knows how to protect against an STI	54.9% (51)	64.0% (25)	46.2% (26)
Adolescents	40.0% (20)	25.0% (8)	50.0% (12)
Adults	64.5% (31)	82.35% (17)	42.9% (14)
Knows where to get an STI exam^8^	96.6% (29)	100.0% (13)	93.3% (14)
Has had an STI	23.5% (51)	40.0% (25)	7.7% (26)
Refused	7.8%	8.0%	7.7%
Adolescents	80% (20)	75.0% (8)	83.3% (12)
Refused	20%	25.0%	16.7%
Adults	38.7% (31)	58.8% (17)	14.3% (14)
Refused	0.0%	0.0%	0.0%
**HPV**			
Knows HPV is a sexually transmitted infection	2.0% (51)	0.0% (25)	3.8% (26)
Knows that HPV can affect:			
Females only	24.0%	45.8%	3.8%
Males only	30.0%	0.0%	11.5%
Both females and males	56.0%	45.8%	65.4%
Don’t know	14.0%	8.3%	19.2%
**Cervical cancer**			
Knows that HPV can cause cervical cancer	49.0%	44.0%	53.8%
Don’t know	39.2%	32.0%	46.2%
Knows that cervical cancer can cause death in women	0.0%	7.9%	0.0%

*Note. HPV = human papillomavirus*.

8 Of those knowing what an STI was (Number knowing what an STI was).

### 3. Willingness to Vaccinate against HPV and Immunization Preferences

Participant willingness to vaccinate and preferences for vaccination are shown in Table 3. All participants had previously received a vaccine, and 100% of participants said they would like the HPV vaccine to be available in Mali. The majority of participants would only receive immunization against HPV if the vaccine were available at no cost to participant (68.6%). One-hundred percent of participants reported being willing to receive HPV vaccination, while only 74.5% were, or would be, willing to vaccinate their child(ren) against HPV.

**Table 3:** Willingness to participate in vaccination.

	%
Ever received any vaccination	100.0%
Would like the HPV vaccine to be available in Mali	100.0%
Willing to receive HPV vaccine	100.0%
Willing to vaccinate child(ren) against HPV^9^	74.5%
Would get vaccinated against HPV if the vaccine cost:^10 11^	
0.18USD	7.8%
0.37USD	9.8%
0.93USD	7.8%
9.27USD	5.9%
Only if the vaccine were free	68.6%
Would prefer to be contacted to receive information about vaccination appointments by:	
Phone call	41.2%
Text message	2.0%
A home visit	21.6%
Public crier	3.9%
Television	2.0%
A message at school	17.7%
Other	11.8%
Would prefer to receive the vaccine at:	
A hospital	22.0%
The local health clinic	46.0%
School	10.0%
Home	22.0%

*Note. HPV = human papillomavirus, USD = United States dollars*.

9 All participants were asked this question. This question was hypothetical for those without children.

10 Percentages do not equal 100% due to rounding.

11 Converted from CFA using an exchange rate of 539.4CFA/USD.

Participants suggested the following methods of contact for receiving information about HPV vaccination appointments: phone calls (41.2%), home visits (21.6%), messages at school (17.7%), public crier (3.9%), text messaging (2.0%), and television messages (2.0%). Participants preferred receiving the vaccine at a local health clinic (46.0%), at a hospital (22.0%), at home (22.0%), and at school (10.0%).

### 4. Autonomy in the Decision to Vaccinate

Ability to autonomously decide to vaccinate oneself against HPV is summarized in Table 4, and was significantly different across female adolescents (15.4%), male adolescents (46.2%), adult female (75.0%) and adult male (76.9%) participants (Fishers exact test p = 0.005). Adult males 76.9%) had 0.176 times the odds of having the ability to vaccinate autonomously than females and adolescent males (44.7%) (95%CI 0.03–0.61; McNemar’s test p = 0.0026). Adult males had 10.0 times the odds of having the ability to autonomously decide to vaccinate their child against HPV (84.6%) compared to adult females and adolescents (64.5%) (95%CI 2.43–88.24; McNemar’s test p = 0.0001).

**Table 4:** Autonomy in the decision to vaccinate.

	Female adolescents	Male adolescents	Women	Men	Fisher exact test p-value
Would decide autonomously to receive HPV vaccine:	15.4%	46.2%	75.0%	76.9%	0.005*
Would decide autonomously to vaccinate child(ren):	33.3%	69.2%	75.0%	84.6%	0.185

*Note. HPV = human papillomavirus.*

* p-value<0.05.

## Discussion

Our results are concordant with the existing literature on HPV vaccine knowledge acceptability in both resource-rich and resource-limited settings, which is that while levels of knowledge are generally low, individuals are willing to receive vaccination against HPV [26]–[30] [38] [35]. We found that knowledge of HPV and cervical cancer amongst participants was low, yet willingness to receive HPV vaccination and willingness to vaccinate themselves and/or their child was high. Our findings also suggest that relying on cellular phones methods for modes of contact is not likely to succeed in Mali, which in 2006 had low cell phone tower coverage of 1.1% of area and 18.1% of population [39]. Moreover, questions assessing preferences on the location for receiving vaccine resulted in sample subset-specific responses; adolescents were more likely to prefer vaccination at school and females to prefer vaccination at home. The selection of sites at which to distribute vaccine may need to be targeted to each eligible subgroup. As the available HPV vaccine is recommended for use in males and females aged 9–26 years, these differences will be critical in developing immunization campaigns tailored to reach both adult and adolescent males and females.

We identified that adult male participants were significantly less likely to require permission from someone else in order to receive the HPV vaccine in comparison to all other sample subsets. Adult males also had higher odds of autonomy in the decision to vaccinate their children than all other sample subsets. Our research is, to the best of our knowledge, the first to study to examine the differences in permission required to receive HPV vaccination across all vaccine-eligible individuals. This information is critical for the development of vaccine campaigns in settings where all individuals do not have equal autonomy.

We identified men as the primary decision makers regarding HPV vaccination, and as such they may be key targets for vaccine campaigns. This attitude suggests that men are important factors in the vaccination of women and adolescents, and better immunization outcomes for women and adolescents may be expected if men are involved in this population [18]. Frameworks for a new reproductive health paradigm highlight the need to incorporate men into emerging immunization programs [40]–[41]. Addressing men as actors in the health decisions of women and children may be more important in the introduction of STI vaccination in predominantly Muslim populations. In a study conducted in Malaysia, a country where Islam is the national religion, men were found to play an influential role in the vaccination decisions of their children and spouses [38]. In this study, men were in favor of protecting their spouses, partners, or daughters from cervical cancer by HPV vaccination after receiving information about HPV infection and vaccination [38]. In Mali, adolescent females may marry as young as 15 years with parental consent [42], and married adolescent females would require permission from their husbands. Opt-out consent for HPV vaccination was considered acceptable in Tanzania [29], and represents a possible vaccination strategy addressing the lack of autonomy female adolescents have in accessing health care.

Vaccinating adolescents before sexual debut was initially found to be challenging in sub-Saharan Africa because of limited health care funding, rare reproductive health services, and dispersed populations [43], however more recent studies indicate that HPV vaccination can be delivered with high coverage and acceptability amongst adolescent females. Our findings on the age of sexual debut are comparable to the results from previous research in Bamako, Mali, finding that 40% of female adolescents and 25% of male adolescents reported having had sex by the age of 15 years [44]. Our study identifies autonomy in the decision to receive vaccination as an additional barrier to the vaccination of women and adolescents in Mali, West Africa. The association between male circumcision and HPV transmission has been characterized; however no studies have examined the relationship between female circumcision and HPV infection in females. The great majority of females in our study population were circumcised, and thus the role of female circumcision in HPV prevalence requires further investigation.

The sample size of this study is not representative, and thus limitations of this research include the precision of the findings which should be interpreted with caution. The unavailability of data on the number of households and the population of each household in Mekin-Sikoro and the convenience sampling of households proximal to the health center are additional limitations to this study. All interviews were conducted in the home, which may have led to social desirability bias, particularly in response to sexual history questions. Nevertheless, this study provides information to guide future representative studies of HPV knowledge and vaccine acceptability in Mali, West Africa. The high rate of willingness to receive HPV vaccination despite low HPV knowledge presents ethical challenges to implementing an HPV immunization program in Mali. Importantly, our results suggest that autonomy in the decision to receive HPV vaccination is not equally distributed in this setting and should be investigated. More research is needed to further characterize decision makers and incorporate this information into targeted HPV vaccine campaigns.
